# Efficient and accurate treatment of electron correlations with Correlation Matrix Renormalization theory

**DOI:** 10.1038/srep13478

**Published:** 2015-08-28

**Authors:** Y. X. Yao, J. Liu, C. Liu, W. C. Lu, C. Z. Wang, K. M. Ho

**Affiliations:** 1 Ames Laboratory–US DOE and Department of Physics and Astronomy, Iowa State University, Ames, Iowa 50011, USA; 2 State Key Laboratory of Theoretical and Computational Chemistry, Institute of Theoretical Chemistry, Jilin University, Changchun, Jilin 130021, China; 3 College of Physical Science and Laboratory of Fiber Materials and Modern Textile, Growing Base for State Key Laboratory, Qingdao University, Qingdao, Shandong 266071, China

## Abstract

We present an efficient method for calculating the electronic structure and total energy of strongly correlated electron systems. The method extends the traditional Gutzwiller approximation for one-particle operators to the evaluation of the expectation values of two particle operators in the many-electron Hamiltonian. The method is free of adjustable Coulomb parameters, and has no double counting issues in the calculation of total energy, and has the correct atomic limit. We demonstrate that the method describes well the bonding and dissociation behaviors of the hydrogen and nitrogen clusters, as well as the ammonia composed of hydrogen and nitrogen atoms. We also show that the method can satisfactorily tackle great challenging problems faced by the density functional theory recently discussed in the literature. The computational workload of our method is similar to the Hartree-Fock approach while the results are comparable to high-level quantum chemistry calculations.

It is one of the outstanding challenges in physics, chemistry, and materials science to develop robust and efficient theoretical and computational methods to accurately calculate the electronic structure and total energy of materials containing strongly correlated electrons (see, for example, US DOE-Office of Science Report: “Basic Research Needs for Advanced Nuclear Energy Systems”, 2006). While accurate methods are available from quantum chemistry approaches (e.g., configuration interaction (CI)), these methods are too expensive for condensed matter systems. On the other hand, density functional theory (DFT) and related computational codes based on the Kohn-Sham approach^1^,^2^ have been well developed, and are highly effective and successful for predicting the structures and properties of many materials, but they fail for systems with strongly correlated electrons. In the last three decades, there have been intensive efforts in developing new approaches to solve the outstanding problems in correlated electron systems^3^,^4^,^5^,^6^,^7^,^8^,^9^,^10^,^11^,^12^,^13^,^14^,^15^,^16^,^17^,^18^,^19^. Among these new developments, local-density approximation plus on-site Coulomb interaction parameter U (LDA + U)^3^,^4^, LDA + dynamical mean field theory^5^,^6^,^7^, and LDA + Gutzwiller^11^,^12^,^14^,^15^,^16^,^17^,^18^,^19^ have emerged as the most popular methods for treating strongly-correlated electrons in solid-state systems. These methods handle electron correlations through the effective on-site Coulomb interaction parameters, while keeping the overall description of the electronic structure through LDA. The effective Coulomb parameters can be calculated using constrained LDA^3^ or constrained random phase approximation^20^. Completely self-consistent approaches are also possible^21^. However, the effective Coulomb parameters can often be treated as fitting parameters in practical calculations, especially when one intends to study the total energy of the correlated systems^6^,^16^.

In this paper, we present a highly efficient method for the electronic structure and ground state total energy calculations of strongly correlated electron systems without adjustable Coulomb parameters. In our approach, the commonly-adopted Gutzwiller approximation (GA) for evaluating the one particle density matrix^22^,^23^,^24^,^25^ is extended to evaluate the two-particle correlation matrix of the system. This approximation, which we call the correlation matrix renormalization (CMR) approximation^26^, allows the expectation value of a many-electron Hamiltonian with respect to Gutzwiller variational wave function (GWF) to be evaluated with reduced computational complexity. We show that the method describes well the bonding and dissociation behaviors of hydrogen and nitrogen clusters in comparison with the accurate and expensive quantum chemistry calculations. Furthermore, some of the most challenging problems faced by Kohn-Sham DFT-based calculations recently discussed in the literature^27^,^28^ can also be readily solved by our method. The method has no double counting issues in the calculation of total energy, and produces the correct atomic limit. The computational efforts involve solving the renormalized HF-like equations with O(N^4^) scaling, where N is the number of basis functions to expand the non-interacting wave function, and optimizing the local configuration weights, which scales linearly with the number of inequivalent correlated atoms and exponentially with the number of local correlated orbitals of each atom.

## Results

We start with the Hamiltonian for an interacting many-electron system in the second quantization form


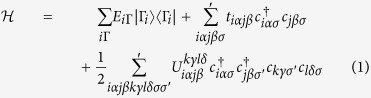


Here 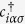 and *c*_*jβσ*_ are electron creation and annhiliation operators, with *i*, *j*, *k*, *l* atomic site indices, *α*, *β*, *γ*, *δ* orbital indices and *σ*, *σ*′ spin indices. The first term of Eq. (1) is the local on-site Hamiltonian which has been singled out for exact treatment, with *E*_*i*Γ_ the energy of a local many-body configuration 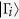. The second and third terms describe the non-local one-body and two-body contributions. All interactions are included in this Hamiltonian without any adjustable parameters. When evaluating this Hamiltonian with the full CI wave function, one obtains an exact expression of the total energy which consists of non-local one-particle and two-particle density matrices in addition to the local on-site contributions. In our CMR approach, we evaluate the Hamiltonian in Eq. (1) with the GWF of the form


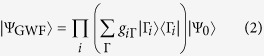


where 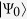 is a non-interacting wavefunction, i.e., a single Slater determinant. *g*_*i*Γ_ is the Gutzwiller variational parameter which determines the occupation probability of the on-site configuration 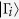. Note that here we restrict the Gutzwiller variational parameters *g*_*iΓ,Γ*_′ to be diagonal in Γ_*i*_, which is still a legitimate choice from the variational point of view and is computationally much more efficient. The central physics of the Gutzwiller trial wavefunction relevant to correlation effects is the selective suppression of the energetically unfavorable atomic configurations in the noninteracting wave function. Using the GWF of Eq. (2) and adopting the generally accepted GA for the expectation value of a one-particle operator^24^,^25^, the total energy of the system within the CMR approach can be casted in a form following the Levy-Lieb’s constrained search method^29^,^30^ as


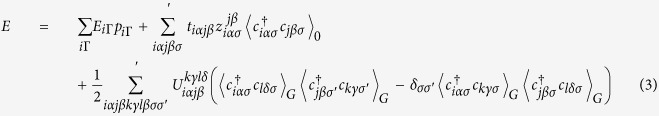


where *p*_*i*Γ_ is the optimized occupation weight of the configuration 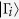. 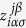 can be evaluated following the standard GA rule for one-particle hopping operators, i.e., 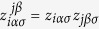 if (*iα*) ≠ (*jβ*) and 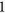 otherwise. Here *z*_*iασ*_ is named Gutzwiller orbital renormalization factor and can be expressed as


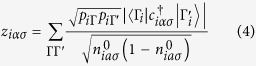


here 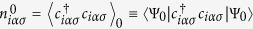. The sum is over all possible local electronic configurations. We define 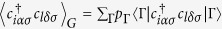 if (*iα*) and (*lδ*) are local correlated orbitals of the same site, and 
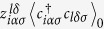
 otherwise. Note that this term effectively adds the crystal field to the local Hamiltonian. To reach the expression Eq. (3), the validity of Wick’s theorem has been assumed. There would be residual correlation energy, *E*_*c*_, to correct the total energy expression in Eq. (3), due to the approximations involved in the CMR approach. In general, *E*_*c*_ can be determined by comparing the total energies from the CMR with that from accurate CI or quantum Monte Carlo calculations for some exactly solvable structures. Since the dominant local onsite electron correlation effect has been taken into account by the GWF, the residual correlation energy due to the CMR approximation is expected to be small. In the test cases to be shown in this work, we find that one way to include the effects of *E*_*c*_ is to modify the renormalization *z*-factor obtained from the GA. Thus, in what follows, the role of *E*_*c*_ is played by replacing 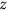 with some functional *f*(*z*), which is determined by fitting on some exactly solved reference systems following the idea of LDA.

**Figure 1:**
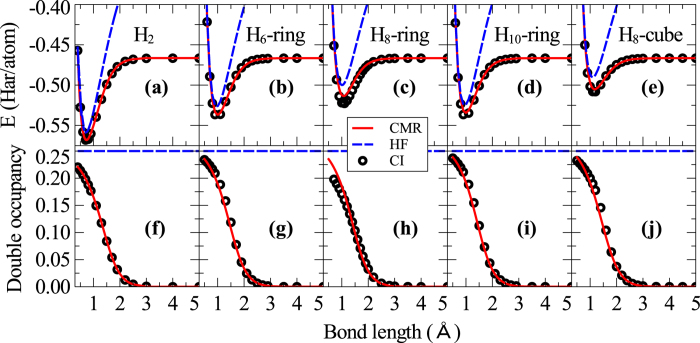
Minimal-basis binding energy and double occupancy curve of hydrogen clusters. The total energy **(a–e**) and double occupancy weight (**f–j**) of H_*n*_ clusters as a function of bond length calculated from the CMR method agree very well with the results from the exact CI calculations. The CMR results are also much better than the HF results. The calculations are done using a minimal basis set.

We first demonstrate the CMR method by studying the dissociation behavior of the hydrogen molecules. The dissociation behavior of these hydrogen molecules has been the testing ground of methods for correlated electron calculations, because the electron correlation changes from the weak to strong regime as the hydrogen bond length increases. For these systems, the residual correlation energy is included by replacing the renormalization *z*-factor^24^ by a functional of *z*, i.e., *f*(*z*). The *f*(*z*) is determined by requiring that the total energy and the probability of the local double occupancy for the hydrogen dimer obtained from CMR to be the same as the exact CI results. In the case of using minimal basis set (one 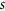 orbital for each H atom), the total energy and double occupancy probability from CI can be calculated rigorously, and *f*(*z*) can be solved analytically in term of *z*. Using this *f*(*z*), CMR calculations are performed on H_6_-ring, H_8_-ring, H_10_-ring and H_8_-cube structures. The results from our CMR calculations are presented in Fig. 1 in comparison with the full CI results. We found the bonding and dissociation behavior of the hydrogen clusters calculated from the CMR method agrees very well with the exact CI results. In contrast, the HF results show large systematic errors, especially at large separations where the electron correlation effect becomes prominent, as evidenced by the strong suppression of the energetically unfavorable local electron double occupancy weight given by CI.

**Figure 2:**
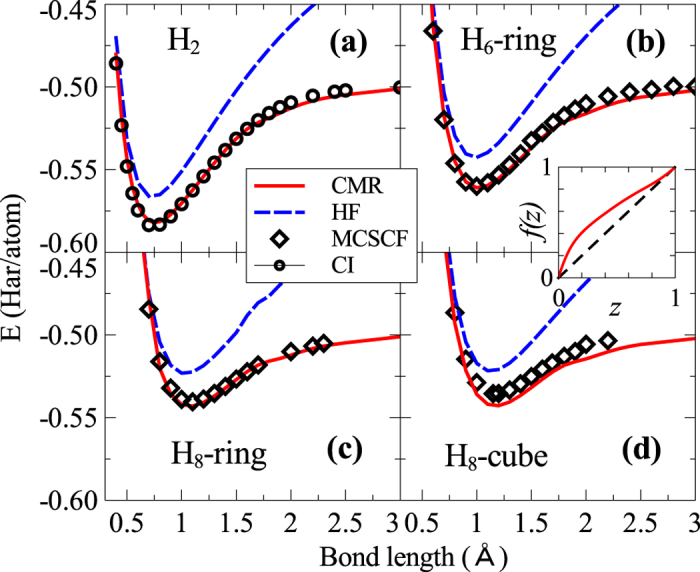
Large-basis binding energy curve of hydrogen clusters. The total energy of hydrogen clusters as a function of bond length calculated from the CMR method agrees well with the result from the high-level quantum chemistry MCSCF calculations. The HF result is also shown for comparison. The calculations are done using large basis set. Insets: *f*(*z*) obtained by fitting the CMR total energy and local configuration weights with the exact full CI results.

We further tested the CMR method on the dissociation behavior of hydrogen clusters using a large basis set of 6-311G**, which contains 3 *s*-orbitals plus 3 *p*-orbitals. In this case, the *f*(*z*) needs to be determined numerically by fitting total energy *E* and {*p*_*i*Γ_} from the CMR calculations on the hydrogen dimer to the exact CI results. Using such a numerically constructed functional *f*(*z*), we have performed the CMR calculations for H_6_-ring, H_8_-ring, and H_8_-cube with the same large basis set. To evaluate the quality of the CMR results, we carried out the highly accurate multi-configurational self-consistent field (MCSCF) calculations for the hydrogen molecules, since the full CI calculations with the large basis set are already very time-consuming. The convergence of MCSCF calculations have been checked such that the error due to truncated active space is smaller than 0.005 Har/atom. In Fig. 2 we show that the CMR method yields again very good bonding and dissociation curves in close agreement with the MCSCF calculations. The inset of Fig. 2 shows the behavior of *f*(*z*), which scales like 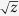 at small *z* and approaches *z* as *z* goes to 1.

**Figure 3:**
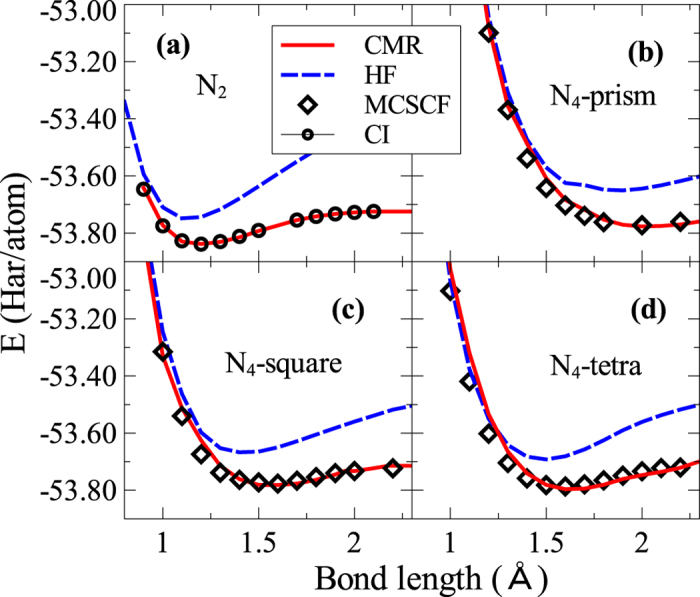
Binding energy curve of nitrogen clusters. The total energy of the nitrogen clusters as a function of bond length calculated from the CMR method agrees well with the result from the high-level MCSCF calculations. The HF result is also shown for comparison.

The CMR method is also successfully applied to systems with atoms containing multiple correlated orbitals, e.g., nitrogen clusters. For computational convenience, we describe the nitrogen atom with the minimum basis set and choose the 2*s* and 2*p* orbitals as the local correlated orbitals. The same idea can be equally well carried over to the large basis calculations as shown previously for the hydrogen clusters. Two functionals, *f*_*s*_(*z*_*s*_) and *f*_*p*_(*z*_*p*_), are introduced to modify the renormalization coefficients of 2*s* and 2*p* orbitals. The specific functional forms, following the procedure in the calculations of hydrogen clusters, are determined by matching the CMR total energy, *E*, and the local configuration weights, {*p*_*i*Γ_}, with the exact CI results of the N_2_ dimer. We apply the method to calculate binding energy curves of three nitrogen clusters of different geometries, i.e., the square, diamond and tetragonal shapes. In Fig. 3 we show the total energy as a function of bond length from the CMR, HF and MCSCF calculations. The good agreement between the CMR and MCSCF energies for all the structures demonstrates the good transferability of our method.

**Figure 4:**
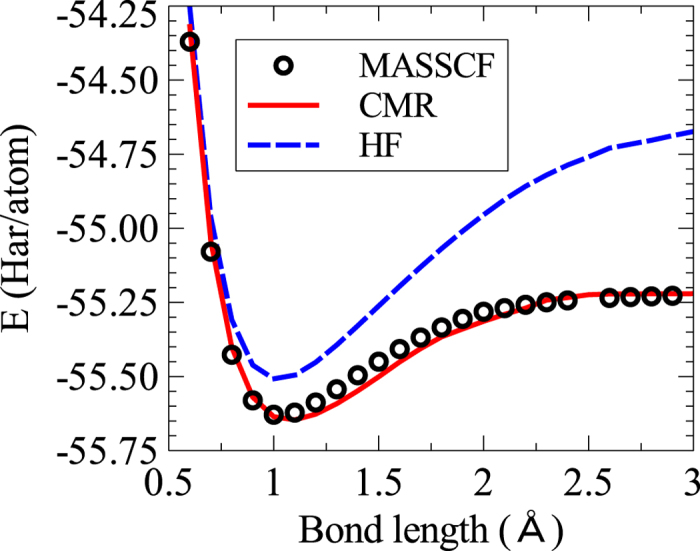
Binding energy curve of ammonia. The total energy of NH_3_ as a function of bond length between H and N atoms is shown in the plot for CMR, MASSCF and HF calculations. H atoms straightly approach the N atom in such a way that the overall stable geometry is preserved.

To further test our scheme, we calculate the binding energy curve of the ammonia molecule, 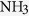, with three hydrogen atoms straightly approaching the nitrogen atom at the apex but maintaining its overall stable geometry. These heterogeneous polarized molecules pose as a very good test bed on the transferability of *f*(*z*) determined with homogeneous molecular dimers. How well these fitted functionals are able to capture the subtle orbital hybridization and correlation energy gain in a chemical process is unknown a priori. We apply the 6-311G** basis set to describe H and the minimum basis set to describe N in order to be consistent with the pre-determined *f*(*z*) for the correlated orbitals in previous calculations. Again, a full CI calculation is time-consuming for this set of basis sets on 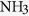. We instead replace it with a highly sophisticated and accurate enough Multiple Active Space Self-consistent Field(MASSCF) method^31^,^32^ to get the benchmark energies with an estimated truncation error smaller than 0.003Har/atom. The calculation is done with GAMESS(US). For MCSCF, a frozen orbital taking 2 core electrons and an active space of 14 molecular orbitals holding the rest 8 electrons are used. For ORMAS-CI, two occupation restricted active subspaces and quadrupled excitations within each active subspace are used. Variationally, more active orbitals and more electron excitations used in MASSCF will render better energy. The current choice is found to balance between speed, accuracy and memory usage quite well. The energy binding curves calculated by CMR, MASSCF and HF methods are shown in Fig. 4. The close agreement between the CMR and MASSCF calculations are easy to see. The HF method, however, gives quite high binding energy curve. This result strongly support the good transferability of *f*(*z*) in different chemical environments.

## Discussion

**Figure 5:**
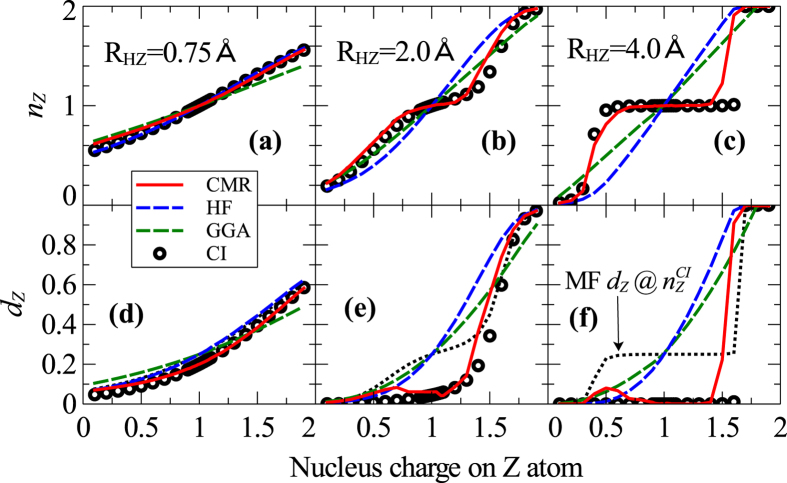
The variation of electron occupation number and double occupancy. The electron occupation number *n*_*Z*_ (**a–c**) and the local double occupancy weight *d*_*Z*_ (**d–f**) as a function of the nucleus charge on the Z atom calculated with the CMR, HF, GGA and CI methods for the HZ dimer at three separations: near equilibrium (left), close to bond breaking (middle) and beyond (right). The dotted line in the lower panel shows the mean field double occupancy weight evaluated at the CI electron occupation of the Z atom.

Very recently, Cohen, *et al.* used some prototype systems to show the dramatic errors in the DFT-based calculations. These errors stem from the fact that the current approximations used in DFT calculations miss the energy derivative discontinuity with respect to the total electron number^27^,^28^,^33^. The prototype systems to reveal the failure of DFT are the stretched few-electron systems, e.g., one-electron systems like HZ^{1*e*}^ and HZH^{1*e*}^ and two-electron systems like HZ^{2*e*}^ with Z being the proton with nucleus charge *Z* varying between 0 and 2. While the electron density from the exact calculations shows dramatic discontinuous changes in real space with a slight variation of *Z* near some critical points at large separations, all the DFT calculations predict an artificial continuous variation of the electron density^27^. Our CMR method gives exact solutions for any single-electron systems, as is easily proved that the orbital renormalization factors are constantly one and the method reproduces the HF results, which are exact in the special class of one-electron systems. Therefore our CMR method yields the exact bonding and dissociation behaviors for both 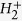 and H_2_ by construction (see Fig. 2), while none of the available DFT calculations can describe both cases equally well^28^. One can further show that because the CMR method reaches the correct atomic solutions at the large separation limit, the exact discontinuous electron transfer observed in the HZ^{2*e*}^ system at large separations can be well reproduced. In Fig. 5 we compare the electron occupation and double occupancy weight of Z atom from the CMR, HF, DFT with the generalized gradient approximation (GGA) and CI. Although all the methods predict similar results near equilibrium bond length (∼0.75Å ), the CMR method shows significant improvements over the mean field HF and GGA and follows closely the exact CI results with increasing separations, even at the chemically crucial bond breaking region (∼2Å ) and beyond. The underlying physics for the large errors of the simple mean field approaches like the HF and GGA can be understood by noting that the mean field double occupancy weight evaluated using the CI orbital occupation, shown as the dotted line in the lower panel of Fig. 5, can severely deviate from the exact CI double occupancy weight—manifesting the multi-configuration nature of the exact solution which is beyond the single Slater determinant description.

**Figure 6:**
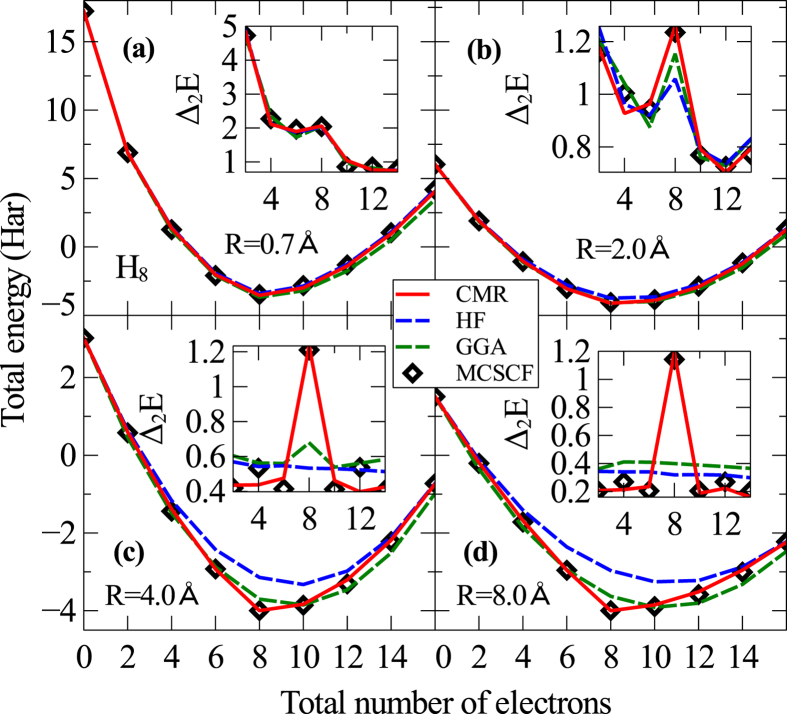
Total energy and its second-order finite difference. The total energy *E* as a function of total number of electrons *N*_*e*_ obtained from the CMR, HF, GGA and MCSCF calculations for the H_8_ cube with increasing bond length *R*. Insets: the corresponding second order finite difference Δ_2_*E*.

Another challenging prototype system is the H_8_ cluster with varying electron filling^28^. The exact solution predicts a relatively big energy gap for the system at large separations and half-filling *N*_*e*_ = 8; while all the DFT calculations fail to reproduce this result because of the incapability to treat the strong electron correlation effects. In Fig. 6 we show the total energy of the H_8_ cube from the CMR, HF, GGA and MCSCF calculations as a function of even number of electron filling, which keeps the system to have the closed shell ground state solution^28^. While all the four theories give similar total energies at the small bond length, the discrepancy becomes increasingly big as the H_8_ cluster expands. Remarkably, the CMR energies agree with the highly accurate MCSCF results very well for all the bond separations and electron fillings, which proves that the key many-body correlation physics in this system has been perfectly captured by the CMR method. A better comparison between the four levels of theories is presented by the energy gap, defined as the second order finite difference 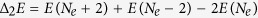, as shown in the insets of Fig. 6. Clearly, as the bond length increases, or the electron correlation effects become stronger, the simple mean field HF and GGA energy gaps show larger deviations from the exact gap, especially the gap at half filling. In contrast, the CMR calculations yield energy gaps in excellent agreement with the MCSCF results in all the cases.

In summary, we have developed an efficient method for calculating the electronic structure and total energy of the systems with strong electron correlations. The method is based on the Gutzwiller type variational wavefunction and adopts a correlation matrix renormalization approximation in which both one-particle density and two-particle correlation matrices at mean field level are renormalized according to the local electron correlation effects. While the computation efficiency of this new approach is similar to the Hartree-Fock method, the calculation results are much more accurate. The benchmark results presented in the current paper for the bonding and dissociation behaviors of the hydrogen and nitrogen clusters show that our method well reproduces the results from the accurate and yet expensive quantum chemistry CI and MCSCF calculations. The calculation applied to ammonia supports the claim of good transferability of CMR from reference homogeneous dimers to heterogeneous polarized molecules. The CMR method is also demonstrated to be accurate for treating the electron correlation effects in some prototype systems where the current DFT and HF calculations fail. The extension of the method to crystalline solids is straightforward and promising, although some additional physics such as screening due to infinite system might cause complications.

## Method

The minimization of the total energy of Eq. (3) with respect to Ψ_0_ and {*p*_*i*Γ_} amounts to solve two coupled eigen-value equations self-consistently^26^. The bare many-electron Hamiltonian is generated by the quantum chemistry code GAMESS^34^. The GAMESS code was also used for the full CI, MCSCF and MASSCF calculations.

## Additional Information

**How to cite this article**: Yao, Y. X. *et al.* Effcient and accurate treatment of electron correlations with Correlation Matrix Renormalization theory. *Sci. Rep.* **5**, 13478; doi: 10.1038/srep13478 (2015).
